# The emergency medical teams initiative in the WHO African region: a review of the development and progress over the past 7 years

**DOI:** 10.3389/fpubh.2024.1387034

**Published:** 2024-06-25

**Authors:** Thierno Balde, Boniface Oyugi, Jerry-Jonas Mbasha, Rashidatu Kamara, Lazaro Gilberto Martinez-Monterrey, Pryanka Relan, Camila Lajolo, Didier Bompangue, Ann Fortin, Joseph Okeibunor, Flavio Salio, Fiona Braka, Dick Chamla, Abdou Salam Gueye, N’Da Konan Michel Yao, Ibrahima Socé Fall

**Affiliations:** ^1^World Health Organisation, Emergency Preparedness and Response Programme, Regional Hub for West Africa, Dakar, Senegal; ^2^World Health Organisation, Emergency Medical Teams Initiative, Geneva, Switzerland; ^3^Centre for Health Services Studies (CHSS), University of Kent, Canterbury, United Kingdom; ^4^World Health Organisation, Emergency Preparedness and Response Programme, Regional Office for Africa, Brazzaville, Democratic Republic of Congo; ^5^World Health Organisation, Addis Ababa, Ethiopia; ^6^University of Kinshasa, One Health Institute for Africa, Kinshasa, Democratic Republic of Congo; ^7^World Health Organisation, Yangon, Myanmar; ^8^World Health Organisation, Department of Strategic Health Operations, Geneva, Switzerland; ^9^World Health Organisation, Global Neglected Tropical Diseases Programme, Geneva, Switzerland

**Keywords:** emergency medical teams, WHO African region, public health, health emergencies, COVID-19

## Abstract

**Background:**

The WHO Emergency Medical Teams (EMT) Initiative coordinates the deployment of qualified medical teams who promptly respond to public health emergencies (PHEs) and provide quality service during emergencies whilst strengthening capacity. Globally, 40 EMTs have been classified between 2016 and the present (as of the writing of this article in December 2023) and are from across all the WHO regions except the WHO Africa Region (AFRO). However, WHO Africa has prioritised the implementation of EMTs in 10 priority countries to address the public health emergencies (PHEs) affecting the region.

**Objective:**

This article describes the development and progress of national EMTs in the WHO African Region over the past 7 years and elucidates the main lessons learned and the complexity and challenges in the process.

**Methods:**

This study employed a case study approach because of its appropriateness in examining a complex social phenomenon in a socio-political context in depth, using multiple lenses simultaneously. Data and information were obtained through document reviews and key informant interviews (KIIs) (*n* = 5) with the members of the EMT Initiative on shared field experiences. Data were systematically analysed using the Stages of Implementation Completion (SIC) framework, and the lessons learnt were presented using components of a framework from Adini et al.

**Results:**

The Initiative commenced in the WHO African Region following its launch in December 2017 in Senegal. The assessments of the concept’s engagement (involved learning and deciding), feasibility (reviewing expectation and capacity), and readiness planning (collaborating and preparing) showed that the context-specific (African context) challenges, lessons from different emergency response actions mainly guided the Initiative’s pre-implementation phase in the region and prompted the WHO emergency leadership on the urgency and need for the EMT concept in the region. The assessment of the implementation processes showed progress in key areas, with staff demonstrating improved competency, EMT services maintaining high fidelity, effective consultation launching critical components, and ongoing services providing successful support and monitoring. Creating the N-EMTs and revitalising the EMT concept required an aligned strategy with other regional emergency programmes and a futuristic vision. Proposed sustainability and governance components include creating N-EMT, developing a coordination structure, collaborating with partners, and finalising the N-EMT.

**Conclusion:**

The Initiative is an imperative component that would allow better-targeted management of health emergencies in the region. The continuous refinement of the EMT initiative is crucial. There is a need to work on additional components, such as a context-specific framework for collaborations and partnerships that would enhance deployment and procurement modalities and the complementarity between other regional initiatives to improve the work. Emphasis should be placed on strengthening local health systems, enhancing training and capacity-building programmes, and fostering regional and international collaborations. Additionally, sustainable funding and resource allocation are essential to ensure the resilience of EMTs in the African region and their long-term success.

## 1 Introduction

Low-income countries (LIC) and low- and middle-income countries (LMIC) are disproportionately burdened by the occurrences of outbreaks, disasters, and other complex emergencies (1). These countries have weak healthcare systems and are substantially strained by a lack of supplies, inadequate infrastructure, and a limited health workforce, which hinders response efforts and the needed quality healthcare when faced with emergencies (2). Furthermore, they are disproportionately affected by poor disaster-preparedness systems and the lack of response resources despite the urgently needed logistical and health requirements during emergencies (3, 4).

The World Health Organisation (WHO) developed the Emergency Medical Teams (EMT) initiative in 2015 (5, 6). The Initiative was built based on the lessons (such as poor coordination of the responses by the medical teams that were largely characterised by an absence of standardised care and accountability and governance) learned from the response experience of the earthquake in Haiti in 2010 (5–7). Subsequently, an expert review was conducted on the Foreign Field Hospitals following the aftermath of sudden-impact disasters (SIDs) by the Pan American Health Organization (PAHO), which, together with the lessons from Haiti, prompted the need for standardisation of response actions and coordination (5, 6). As a result, the WHO launched a series of steps to develop the criteria, principles, and standards for medical teams and the global EMT registry (1, 8). The registry envisaged having EMTs voluntarily apply to undergo a classification process of EMTs to demonstrate that they meet the internationally accorded EMT minimum standards and agree to provide quality care using accepted guidelines. After the classification, the EMTs would then be deployed to respond to emergencies with the support of WHO and following a request of the host government (1, 8).

The EMT Initiative coordinates the deployment of qualified medical teams who promptly respond to emergencies (such as conflicts, climate-related disasters, and outbreaks) and provide quality service during emergencies whilst strengthening capacity and fortifying overwhelmed health systems (9, 10). Additionally, the Initiative focuses on training for specialised and general emergency disasters and diseases and supports governments in developing policies towards establishing EMTs. Over the last 10 years, there has been an improvement in the standardisation and professionalisation of care for emergency-affected communities, partly because of the changes and initiatives spearheaded by the WHO EMT initiative (4). The Initiative has provided a platform for developing specialist EMTs and strengthening specific emergency health and logistical responses. One critical focus of this Initiative has been building National EMTs (N-EMTs) within the countries in the continent and enhancing and strengthening their capacity. N-EMTs are healthcare professionals organised at the national level to provide rapid medical care during emergencies (such as natural disasters, disease outbreaks, or humanitarian crises) and who are quickly deployable to affected areas, providing critical healthcare services and supporting overwhelmed local health systems. The Initiative emphasises having teams of appropriately skilled personnel who provide care in a coordinated approach that targets the health needs during an emergency whilst meeting the minimum standards of care (4).

Globally, 40 EMTs have been classified between 2016 and the present (as of the writing of this article in December 2023) and are from across all the WHO regions except the WHO Africa Region (AFRO) (11). However, some WHO AFRO countries have begun building capacities and garnering resources to establish their N-EMTs to address the public health emergencies (PHEs) affecting the region. Nonetheless, given the heterogeneity in characteristics in the different countries in the continent and the pre-existing vulnerability of the populations, the context-specific of every emergency (such as their variation based on scale and the capacity of the affected community and country to manage the event) (2), the response strategies needs to be tailored to the specificity.

This article describes the development and progress of EMTs in the WHO African Region over the past 7 years. It further elucidates the lessons learned, including the complexity and challenges, and proposes recommendations for improving EMT work.

## 2 Methods

### 2.1 Analytical framework

The implementation processes of interventions are designed to be flexible, capturing potential variations. This adaptability ensures that assessment measures can inform progression through implementation stages, increasing the likelihood of success (12, 13). The process evaluation of interventions in implementation science involves analysing the formulation and implementation (including the role of different actors, processes involved, the fidelity of implementation, and the nature of the contexts) (14, 15). In this study, we systematically document and describe the formulation and implementation (development and progress) of EMTs in the WHO African Region over the past 7 years using the Stages of Implementation Completion (SIC) framework (16). SIC framework outlines three phases: pre-implementation, implementation, and sustainment, further divided into eight implementation activities: engagement, consideration of feasibility, readiness planning, staff hired and trained, adherence monitoring processes in place, services and consultation begin, ongoing services, consultation, fidelity monitoring and feedback, and competency. In our study, we chose the model to fit the distinct lessons, perspectives, and timelines of implemented activities following a standard sequence of steps reminiscent of an implementation cycle, and we summarise the activities necessary to move towards successful programme start-up, competency, and sustainment (see Table 1). On the other hand, the lessons learnt were presented using components of a framework from Adini et al. (17), as summarised in Table 1.

**Table 1:** Implementation phases, stages, and processes/actions, and lessons learnt.

Phase	Stage	Actions and timelines	Lessons learnt
	Stages of implementation completion (SIC) framework		Framework by Adini et al.
Pre-implementation	Engagement Consideration for feasibility Readiness planning	**2015–2017:** the regional teams’ engagement and feasibility testing of the concept happened based on the continent’s challenges and lessons learned from previous responses. **2017–early 2019:** the concept’s readiness planning (collaborating and preparing) was done through regional sensitisation workshops and country awareness campaigns.	Financing and operations supply and logistics (OSL) Initiation and driving desire Planning, regional, and global collaboration Human resource challenges and opportunities Training programmes and exercises and contextualising the concept
Implementation	Staff Processes in place Services Fidelity, monitoring, and feedback	**2018–2019 and 2022–2023:** launching critical components, such as the development of N-EMTs and deployments, enhanced staff skills, and knowledge transfer. **2020–2022**: the COVID-19 pandemic enhanced the EMT implementation aspects of services support and monitoring and staff support, fortifying the development of the N-EMTs in the region. **2020–2023**: establishment of the Regional EMT Training Centre and EMT training.	
Sustainability	Competency	**2022 to date:** developing the WHO strategy and course of action for the future. Implementation of N-EMTs in the proposed priority countries. Steps and roadmap for developing N-EMT in the priority countries (10 steps for building N-EMTs) Proposing establishing the regional EMT governance structure. Working on a linkage between the EMT and other regional initiatives.	

### 2.2 Design

In this study, we employed a case study approach because of its appropriateness in in-depth examining a complex social phenomenon in a socio-political context, using multiple lenses simultaneously (18–20). In this design, the case described is the EMT initiative in the WHO African Region, whilst the focus or the unit of analysis is the Initiative’s formulation and implementation. Furthermore, the temporal boundaries are the past 7 years during which the Initiative has been implemented, whilst the case parameters are those the Initiative has supported in the context.

### 2.3 Data collection and analysis approaches

Data and information used in this study were obtained from document reviews and key informant interviews (KIIs). These approaches were based on the methodologies applied in previous studies that focussed on the deployments of international EMTs (I-EMTs) (5, 21–24).

For the KIIs, we considered respondents who were knowledgeable about the EMT initiative and had been involved in the formulation and entirety of the implementation. In this study, we refer to them as insiders. Insider researchers are considered to have a place in a social group being studied (25, 26). They have been described as those who can have a more authentic understanding of the culture and are truthful with the concepts being studied, given their easy access to the context and concept being studied through their first-hand implementation experience (27). However, researchers have shown they can also be inherently biased (28). In this study, they elucidated insights into the dynamics, processes, and approaches employed in the Initiative’s implementation in the region and highlighted the lessons learned and challenges from the implementation. As part of their roles in the EMT initiative, they either participated in meetings with the different actors such as government officials, EMT teams, and WHO country/regional/headquarters leaders or led different components of the Initiative at different times, which they used for understanding the dynamics of the stakeholders involved in the implementation of the EMT initiative. Five KIIs were interviewed between October and November 2023, with the guides focussing on the evolution of EMT development in the WHO AFRO and the processes involved.

For the document reviews, we used a rapid review approach to assess the elements already known about the EMTs initiative in the WHO African Region over the past 7 years. The approach used systematic reviews to search for and critically appraise the existing evidence (29). It included documents or studies written in English, French, and Portuguese and related to the EMT work (reported the concepts of formulation of the implementation of EMTs) in the WHO African Region. The documents considered in this study were from (a) secondary data availed by the WHO teams in the African Region and headquarters; (b) pivotal websites of the United Nations Office for the Coordination of Humanitarian Affairs and WHO EMTs; (c) Secondary data provided by different countries that have implemented different elements of EMT work (such as EMTs assessments and surveys, mission reports, bulletins, and meeting minutes of key meetings on the EMTs initiative and networks from the different countries in the continent); (d) Direct contact with the deployed I-EMTs in the continent requesting data on their deployments; and (e) Internet searches (Google scholar and PubMed using the search words “emergency medical team OR Emergency medical teams OR EMT,” AND “Africa OR sub-Saharan Africa OR) [all countries in WHO African Region].” The search strategy used for PubMed is detailed in Appendix 1. When searching on Google Scholar, we used the words “emergency medical teams in Africa” and manually reviewed the articles on the first 50 pages. In both sites, we considered articles that met specific criteria, such as language (English, French, and Portuguese), relevance to implementing EMTs, and publication date (from 2017 to the present). Our search on PubMed yielded 845 studies, whilst Google Scholar produced 50. After reviewing them for eligibility, we excluded studies irrelevant to EMT work. We screened all titles and abstracts, identified 19 studies that met our criteria, and included them in the final review. To analyse the content, we used thematic analysis guided by Braun and Clarke (30) to address questions about the journey, lessons learned, and challenges.

## 3 Results

The results are presented in two sections. Section 1 presents the journey and processes the EMT concept has undergone from 2015 to date (as of the writing of this publication in December 2023) using dates and events that happened guided by the stages and actions based on the SIC framework whilst Section 2 presents the lessons learnt using themes based on the framework proposed by Adini et al. (16, 17).

### 3.1 Pre-implementation (engagement, consideration for feasibility, readiness planning)

The assessments of the concept’s engagement (involved learning and deciding), feasibility (reviewing expectation and capacity), and readiness planning (collaborating and preparing) showed that the context-specific (African context) challenges, lessons from different emergency response actions mainly guided the Initiative’s pre-implementation phase in the region and prompted the WHO emergency leadership on the urgency and need for the EMT concept in the region. Examples are discussed in detail below.

#### 3.1.1 2015–2017: the regional teams engaged in and conducted feasibility testing of the concept based on the continent’s challenges and lessons learned from previous responses

Prior to the commencement of engagement with the EMT concept in the region, the lessons learned from responding to global emergencies had led the global EMT initiative to undergo the standardisation of EMT principles. These principles were then applied to different global emergency responses, especially building on the lessons from the West African Ebola outbreak (2014–2016) that had demonstrated the EMTs’ value in outbreak response and other emergencies. The outbreak had the largest deployment of EMTs, involving 58 teams (6). The continent was one of the last to implement EMTs (despite facing numerous emergencies), prompting the need for stronger EMTs in the WHO’s African region and, hence, the beginning of testing the Initiative’s feasibility in the region.

Before the engagement and feasibility testing of the concept in the region, the developed global principles had emphasised the focus of the EMTs as mainly being on trauma and surgical care in response to sudden-onset disasters (SOD). However, after the lessons, broader definitions and EMTs were expected to care for various conditions, from communicable to non-communicable diseases. Additionally, teams were needed to support populations affected by flood, conflict, and protracted crises such as famine, with a need for clinical surge capacity in all emergencies with health consequences (6). EMTs also had a role in reestablishing and maintaining essential health services, taking the discussion of EMT implementation in the region to a new level.

At the onset of the engagement process with the regional WHO African team, the global WHO Health Emergencies (WHE) Programme had just been setup (following the lessons learnt from the response to the Ebola outbreak in 2014–2016 in West Africa) with a philosophical approach of having one programme (at the global, regional and country offices with one budget and workforce) managed from the WHO headquarters (HQ) and with one line of communication (31). The aim was to work with the continent’s member states to augment their capacity to detect, prepare for, prevent, and respond to health emergencies through streamlined emergency response efforts. There were five pillars: preparedness, infectious hazard management, response, health information, and management and administration.

Subsequently, to support the partnership component of the new WHE programme, the team implemented the WHO Global Partnership networks comprising the Global Outbreak Alert and Response Network (GOARN), health clusters, and EMTs. In 2017, the network leadership, having seen the progress in the other WHO regions, had a desire to introduce the EMT initiative in the WHO African region given the challenges with the emergencies the region was facing at the time and allocated funds to the WHO Regional Office for Africa (AFRO) develop the EMT initiative to test the feasibility of the process and support the readiness planning. One respondent noted:

…they allocated USD 300,000 to the WHO Regional Office for Africa (AFRO) to implement strategies for bringing partners together and managing emergencies through [the use of] EMTs. (Respondent 01)

#### 3.1.2 2017–early 2019: the readiness planning (collaborating and preparing) of the concept was done through regional sensitisation workshops and country awareness campaigns

The funds allocated to introduce the EMT concept in the region were used for the initial regional sensitisation workshops in Senegal in December 2017, which formed the basis for the readiness planning. It enhanced the collaboration and prepared the continent to implement the concept. All 47 countries in the WHO African Region received an invitation sent through their WHO country offices (WCOs). 11 of the 47 countries (Cameroon, Burkina Faso, Côte d’Ivoire, Kenya, the Democratic Republic of the Congo (DRC), Nigeria, Madagascar, Rwanda, Senegal, South Africa and Uganda) expressed interests and attended the meeting, and mainly guided by their capacities, history with natural disasters, and potential for better development of EMT (32).

Additionally, regional partners such as Save the Children, the West African Health Organization (WAHO), and the International Federation of Red Cross and Red Crescent Societies (IFRC) also joined. In the workshop, a need for cultural awareness of the EMTs was emphasised, leading to cross-country awareness campaigns (33). Following this recommendation, an awareness workshop was organised across different countries, starting with Senegal in April 2018 (32). The awareness campaign galvanised action around boosting countries’ preparedness for emergencies, advancing the timely deployment of EMTs during emergencies, and harmonising the EMTs’ adherence to minimum standards (34, 35).

A second regional meeting, which laid the foundation for the national implementation plans, was organised in June 2018, jointly with the WAHO in Côte d’Ivoire. It included Burundi and 12 countries of the Economic Community of West African States (ECOWAS): Burkina Faso, Benin, Côte d’Ivoire, Cabo Verde, Ghana, Guinea, The Gambia, Guinea-Bissau, Nigeria, Liberia, Sierra Leone and Togo. More awareness workshops were conducted in 2018 in South Africa (June 2018). The additional ones in Nigeria (October 2018), Guinea (November 2018) and Ghana (November 2018) were jointly undertaken with WAHO. Equally, two independent organisations, Médecins d’Afrique (MDA) and the Alliance for International Medical Action (ALIMA), also participated (32).

From the more awareness workshops, Senegal, South Africa, and ALIMA signed up to undergo the international EMT (I-EMT) classification (recognised international EMT response organisations, whose classification process are carried out annually to discourage unannounced arrivals at emergencies and encourage joining recognised organisations) (35). There was a consensus that countries needed to strengthen their disaster risk management and health emergency capacities urgently. Furthermore, it was resolved that member states needed to adapt EMT’s core and technical standards to fit their country’s context for better preparations to respond to outbreaks and other PHEs. It resulted in a roadmap for institutionalising N-EMT in the countries. As a result, the countries’ capacities were assessed to strengthen them based on the key components of readiness using the EMT Minimum Standards guide (9). In early 2019, an additional EMT national awareness workshop was conducted in Kenya (32).

### 3.2 Implementation staff (support), fidelity monitoring (feedback), consultation (launching of critical components), and ongoing services (support and monitoring)

The assessments of the implementation processes, including staff (support), fidelity monitoring (feedback), consultation (launching of critical components), and ongoing services (support and monitoring), showed progress in key elements. Specifically, the staff demonstrated improved competency, the fidelity of EMT services was maintained at a high level, the consultation process was effective in launching critical components, and the ongoing services successfully provided support and monitoring. Examples are discussed in detail below.

#### 3.2.1 2018–2019 and 2022–2023: the launching of critical components, such as the development of N-EMTs and deployments, enhanced staff skills, and knowledge transfer

Following the awareness workshops, some participating countries launched the critical components of EMT, such as the development of N-EMTs and deployments to tackle growing regional emergencies. For instance, ALIMA, in a collaborative effort with the MoH of DRC, WHO, IMC, IRC, and other development partners, was deployed to support the Ebola outbreaks in May and August 2018. This partnership successfully set up an eight-bed Ebola treatment centre (ETC) at the epicentre of Ebola in the Itipo region, Equateur Province, and a 60-bed ETC in the Beni region of North Kivu Province (36). These ETCs served as crucial points for providing clinical care interventions, screening and isolating suspected cases, and reaching out to neighbouring communities and health facilities. This collaborative effort not only facilitated a significant transfer of skills and knowledge but also improved the case management of patients, laying a solid foundation for the future development of the N-EMT (32).

In October 2018, the Senegal EMT, with the support of the country’s army, was deployed to manage cases in the DRC after a collision between a bus and an oil tanker resulted in a fire that left over 50 dead and more than 100 with severe burns (32, 36). This was done in partnership with the MoH and the WHO. The team provided patient care and worked with local responders to enhance clinical burn care capacity. Following this successful mission in the DRC fire tragedy, the EMT was deployed to Sierra Leone (under WHO’s facilitation) in November 2021 to support the MoH in providing care to the injured after a road traffic accident led to a fire, resulting in over 101 deaths and about 123 injuries (37). The team’s efforts resulted in 87 out of 155 patients being discharged home following successful provision of care (including pain management, reconstructive surgery, palliative and wound care, psychosocial counselling, rehabilitation, and physiotherapy) (38). Furthermore, in August and September 2022, the Senegal EMT, in collaboration with the MoH of The Gambia, responded to the Acute Kidney Injury (AKI) event in The Gambia. This followed The Gambia’s MoH identification of 82 confirmed AKI cases amongst children who were < 8 years old, which had resulted in 70 deaths, with an estimated case–fatality ratio of 85% (39). The WHO, collaborating with the MoH, issued a medical product alert on four possible contaminated cough syrups suspected to have caused the emergency, provided the technical and financial support for the deployment, and conducted an epidemiological study on the same (39).

In August 2023, the MoH Malawi, with support from the WHO AFRO, deployed a national 12-member EMT (from different countries) to Chikwawa District, having been trained in April and June of the same year for preparedness for potential PHEs. The deployment was mainly aimed at enhancing the case management of cholera cases that were on the rise across the country’s districts. They helped mentor the in-house teams in the Chikwawa cholera units, offered case area-targeted interventions/strategies that included surveillance support, shared behavior change messages (by working closely with communities and officials), and distributed essential WASH resources. The different experts in the team minimised the mortality gap by improving the quality of patient care, documenting the processes, supporting the improvement of the guidelines, and working closely with the local team and the communities (40).

#### 3.2.2 2020–2022: the COVID-19 pandemic enhanced the EMT implementation aspects of services support, monitoring, and staff support, fortifying the development of N-EMTs in the region

**Figure 1:**
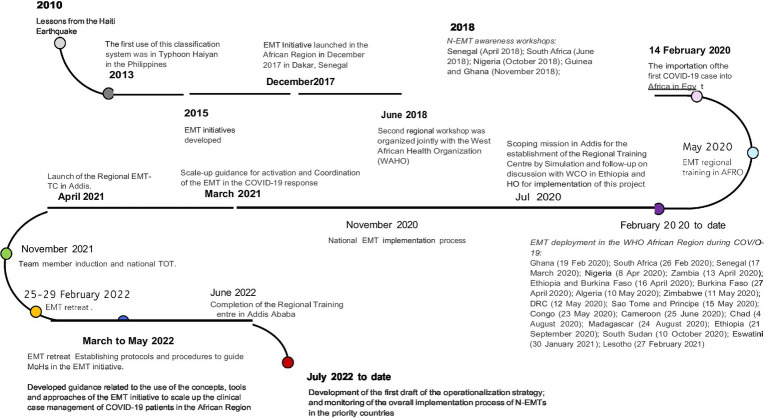
Timelines of the EMT activities in Africa.

The emergence of COVID-19 brought about significant economic and health challenges. Subsequently, through the collaboration of various WCOs and the EMT network, the WHO African Region supported 22 I-EMT deployments in 17 countries, as illustrated in Figure 1 (41). These deployments, initiated in response to requests from the countries for external assistance (based on their diverse needs to address the pandemic at that time), enhanced the EMT implementation aspects of services support and monitoring and staff support, fortifying the development of the N-EMTs in the region. For example, some deployed EMTs stayed longer in certain countries during the peaks of different COVID-19 waves, facilitating coordination and development of guidance on using EMT approaches, concepts, and tools to enhance clinical case management. This also contributed to capacity building for the N-EMTs or teams working to reduce COVID-19 cases.

#### 3.2.3 2020–2023: establishment of the regional EMT training centre and EMT training

Implementing and establishing self-sufficient N-EMTs that adhere to minimum standards, enhance staff capabilities, and ensure service provision has been a priority/vision for the EMT network. As such, the WHO established a regional EMT training centre in Addis Ababa, Ethiopia, which was inaugurated in 2021 and presented a remarkable opportunity to build and enhance in-country capacity and for use for capacity-building activities to complement countries’ process towards developing N-EMT and promoted south-to-south cooperation and reverse innovation (42). The centre offers 10-step training for building N-EMTs, EMT induction training, and EMT field deployment training. These trainings have been organised in collaboration with WHO AFRO, WHO HQ EMT secretariat, and partnering I-EMTs. The centre has also been instrumental in drafting a functional chart of the proposed N-EMTs and setting standard operating procedures (SOPs) for the N-EMTs before and during deployments.

**Figure 2:**
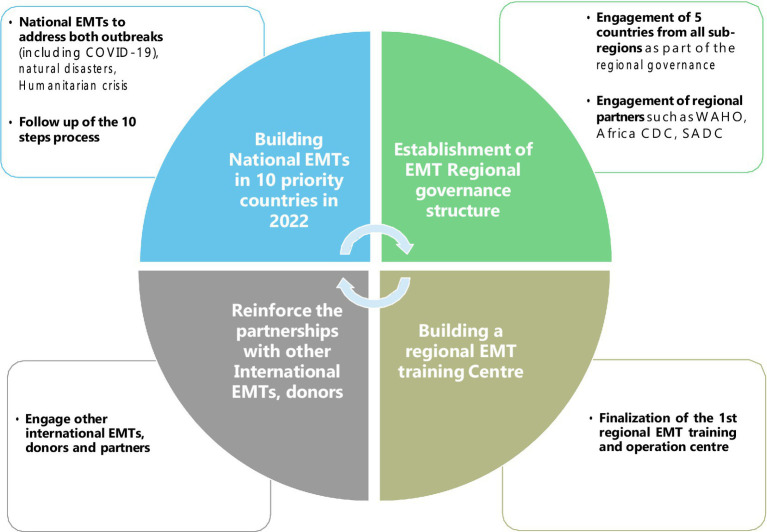
Priority countries for N-EMTs in Africa.

All 10 countries identified for prioritisation in the N-EMT programme (discussed in Section 3.3.1 and Figure 2) have completed the proposed training process. Additionally, countries like Malawi and Mali, which had not initially been prioritised to implement the N-EMT, have also undergone training due to increased response needs (see Table 2). Furthermore, Ethiopia has had four deployments within the country to address issues such as malnutrition, drought, internal conflict, and international response efforts in Turkey and Chad. Senegal has similarly responded to emergencies in other countries, including the burn tragedies in Sierra Leone and the DRC.

**Table 2:** Training that the countries have undergone.

Year	Month	Location	Course type
2020	May	Ethiopia	Team member induction
2021	December	Uganda	Team member induction
2021	December	Uganda	Training of trainers
2021	December	Namibia	Team member induction
2022	February	Ethiopia	Training of trainers
2022	March	Ethiopia	Team member induction
2022	March	Ethiopia	National EMT implementation workshop
2022	June	Ethiopia	Team member induction
2022	July	Ethiopia	Team member induction
2022	October	Mauritania	Team member induction
2022	November	Ethiopia	Massive afflux burns
2022	November	Togo	Team member induction
2022	December	Democratic Republic of the Congo	Team member induction
2023	January	Namibia	Team member induction
2023	March	Malawi	TM induction/cholera CM
2023	June	Malawi	10-step implementation
2023	January	Namibia	10-step implementation
2023	February	Botswana	Team member induction
2023	July	Uganda	Team member induction
2023	July	Uganda	Team member induction
2023	July	Uganda	Team member induction

### 3.3 Sustainment (competency): developing the who strategy and course of action for the future (2022 to date)

**Figure 3:**
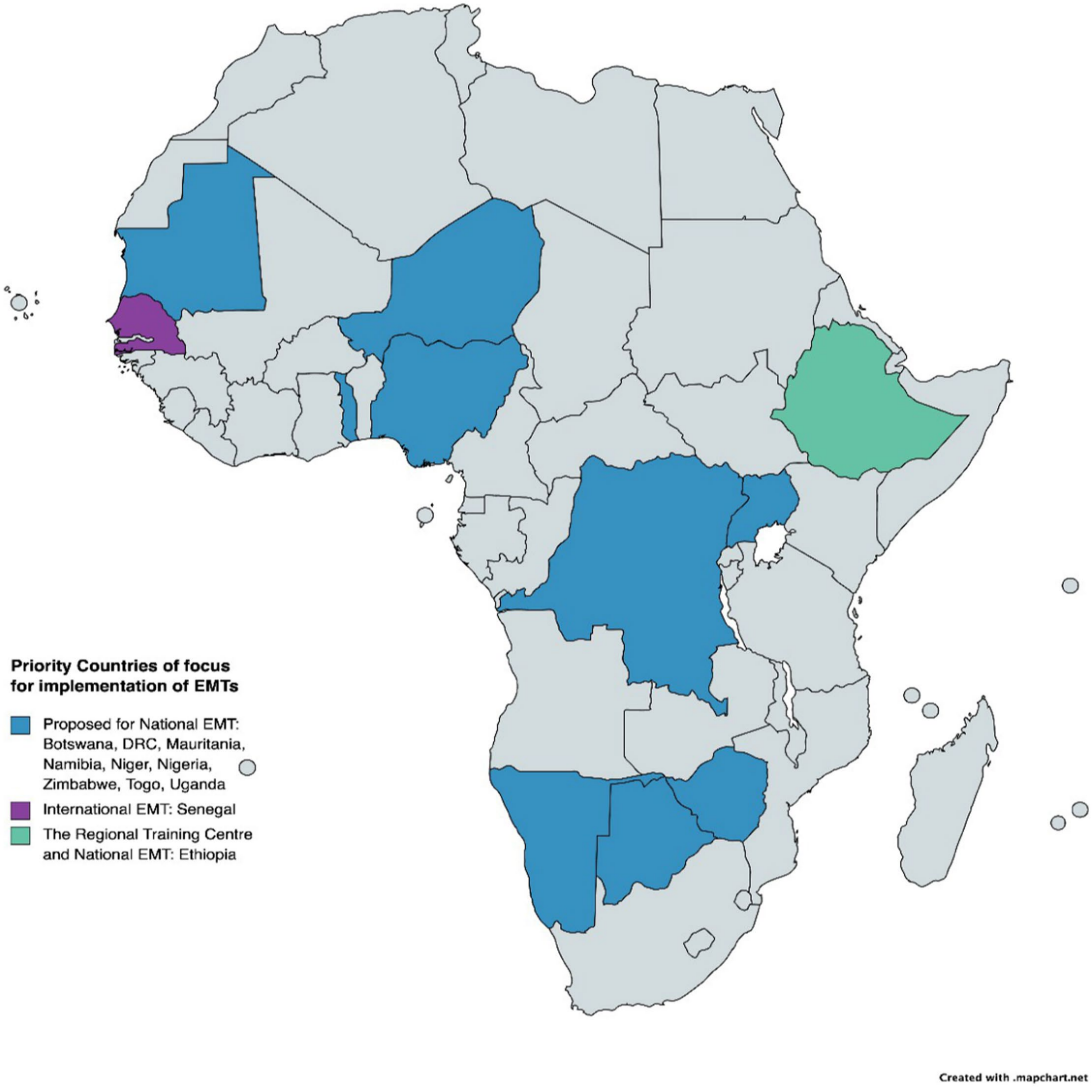
Proposed EMT strategic focus for the WHO African region in 2022. Source: adapted from the regional EMT strategic paper (43).

The creation of the N-EMTs and reinvigoration of the EMT concept in the region required an EMT strategy that would align with the other regional emergency programmes and conceive a futuristic vision for the EMT work in the region. To accomplish this, experts involved in various EMT initiatives, including classification, deployment, management, and research, convened at an EMT retreat in Brazzaville, Congo, in February 2022 to plan for the sustainability and advancement of the WHO strategy and future course of action. The proposed components for sustainability and governance orientations are outlined in Figure 3 and include the steps of creating N-EMT, developing the coordination structure, collaborating with partners, and finalising the N-EMT.

#### 3.3.1 Implementation of N-EMTs in the proposed priority countries

The EMT retreat highlighted the need to establish N-EMTs by collaborating with governments to address emergency challenges, such as logistical challenges during deployment because different countries have specific differences (43). Ten countries [one in Central Africa (DRC), four in West Africa (Mauritania, Nigeria, Niger, Togo), two in East Africa (Uganda, Ethiopia), and three in Southern Africa (Botswana, Zimbabwe, Namibia)] (Figure 2) were prioritised for developing the N-EMT, and Senegal was chosen for implementing an I-EMT. Ethiopia was proposed as the location for the regional training centre.

#### 3.3.2 Steps and roadmap for the development of N-EMT in the priority countries (10 steps for building N-EMTs)

**Figure 4:**
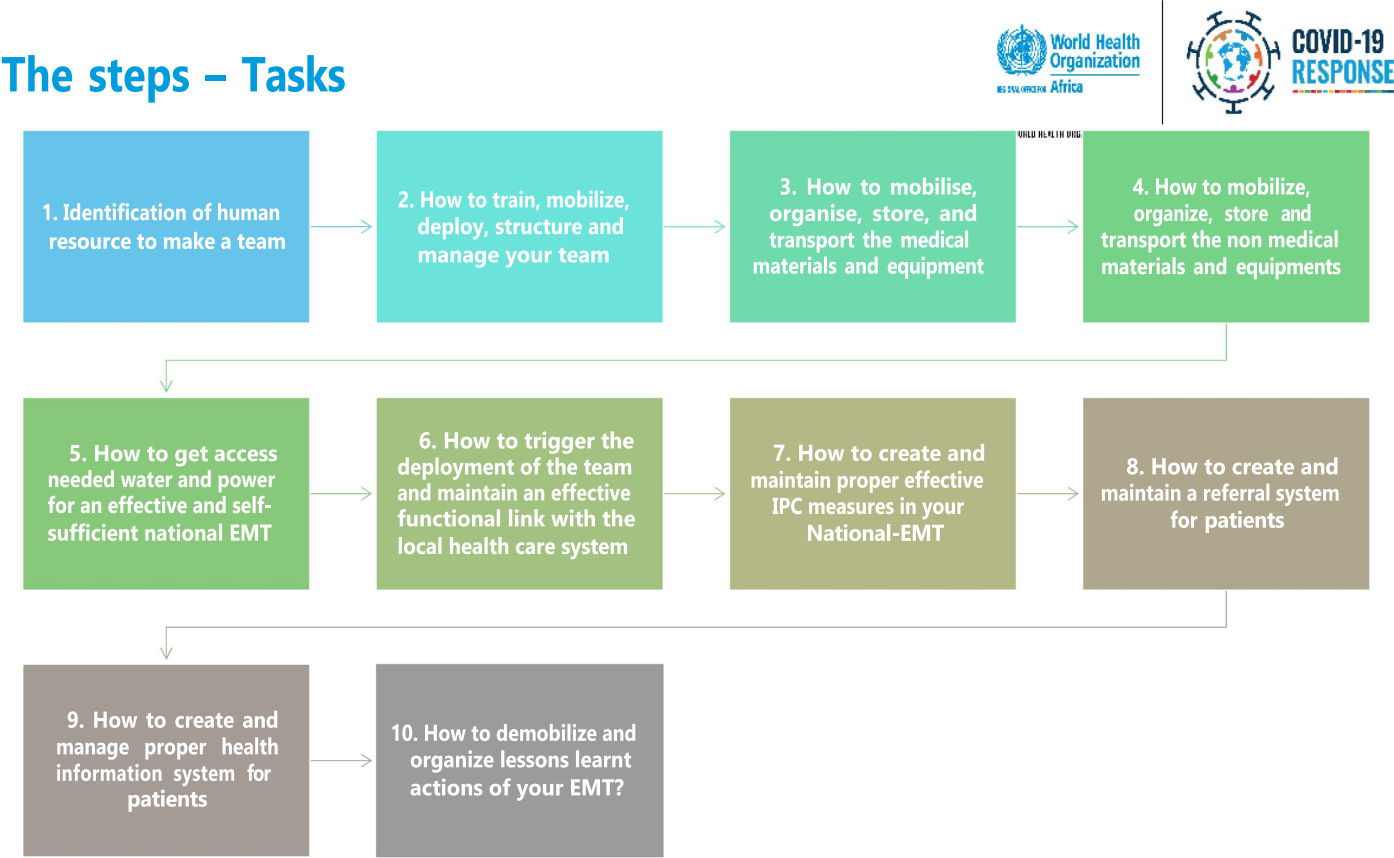
Roadmap for building N-EMT in the priority countries in the WHO African region.

Based on the consensus of experts at the EMT retreat in Brazzaville, Congo, and a two-step literature review process conducted by the team (44), WHO AFRO developed a roadmap to guide N-EMT development in priority countries. This roadmap was based on a curriculum developed by WHO HQ and lessons learned from African emergency response contexts. The steps included forming a core human resource team, training them, managing logistics, knowledge management, and demobilisation (Figure 4). These activities were piloted during the development of the first N-EMT in Ethiopia in April 2022. It was observed that there was variation amongst countries regarding the public health emergencies they face, their context, and the governance system needed to manage the steps. As a result, each country has been redesigning the steps based on its institutional capacity, support, types of public health emergencies, and implementation priorities.

#### 3.3.3 Proposing the establishment of the regional EMT governance structure

**Figure 5:**
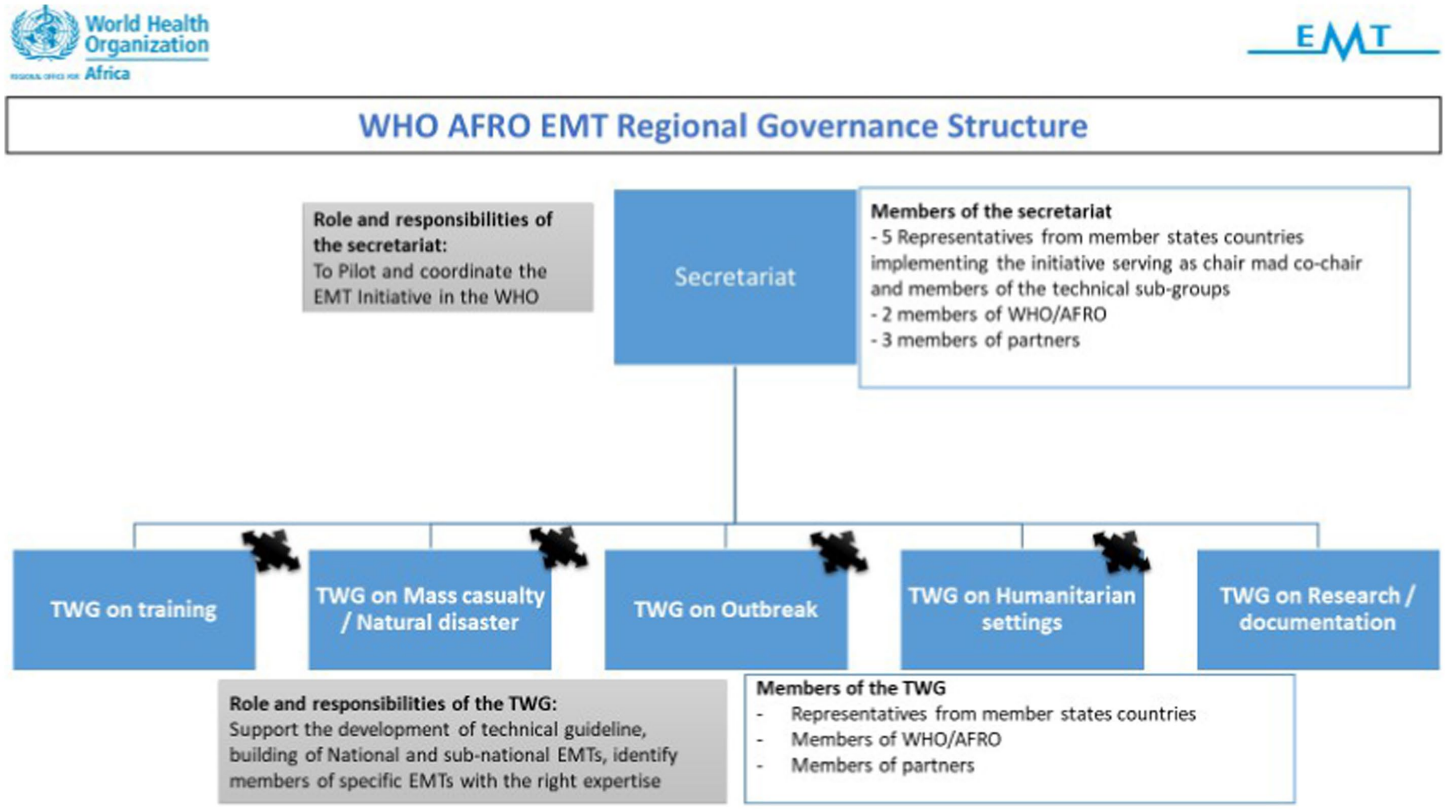
Proposed governance structure of the technical working group (TWG) of the WHO AFRO EMT initiative.

In the abovementioned retreat, the team proposed a regional governance structure envisaged to comprise representatives from countries, partners, and WHO AFRO, as shown in Figure 5 (43). As conceived, it was envisaged that one country would assume a leadership role of ensuring EMT-related information would reach the right people in the countries and participating organisations. Each of the countries would rotate in the position yearly. Five countries (Botswana, Ethiopia, DRC, Mauritania, and Senegal) at an advanced stage in developing their N-EMT at the time volunteered to form the initial governing structure. The EMT representatives from the EMT regional groups were proposed to act as team leaders and the policy and technical focal points of the EMTs in both countries and organisations. It was envisaged and designed that the EMT Regional Group for Africa would facilitate how the countries would participate actively in driving the desire amongst other interested countries and stakeholders and drive the Initiative’s implementation. It would act as a forum to discuss and formulate a regional work plan that would adapt the global objectives of the Initiative to the contexts of the region and countries. In addition, it would provide the strategic orientation of the Initiative. Partners (such as PCPM, MSF, ALIMA, IFRC, CADMEF, and WAHO) were proposed to form part of the key working groups. Other partners would be identified based on expertise and interest in the Initiative. The representatives of the partner would enhance the linkage of the partners and proposed regional groups and learn from their knowledge and experiences.

#### 3.3.4 Working on a linkage between the EMT and other regional initiatives

In implementing the N-EMT, there has been a reflection on aligning with other regional strategies. For instance, through the collaborative project of the WHO AFRO and Africa CDC called the Emergency Preparedness and Response Flagship Initiative (45), the collaborative elements aim to work on Transforming African Surveillance Systems (TASS) Flagship Project, Strengthening and Utilising Response Groups for Emergencies (SURGE) and Promoting Resilience of Systems for Emergencies (PROSE) Flagship Project. The Initiative aims to build on existing systems and efforts such as EMTs, Field Epidemiology and Laboratory Training Programs (FELTP), and Public Health Emergency Operation Center (PHOEC) to achieve its overall objectives of timely response to emergencies, thereby mitigating catastrophic loss to infrastructure and resources and saving lives.

EMT initiative strengthens a resilient health system in the same spirit as the SURGE initiative, and integrating it as a component of the SURGE initiative helps to maximise the capacities, thereby bolstering the flexibility and independence of the countries’ health system capacity to respond and control emergencies in a coordinated, integrated, effective and efficient approach utilising skilled, trained and equipped workforce. Additionally, the EMT initiative fosters knowledge sharing and strategic partnerships with other networks and partners targeted by the SURGE Flagship. Therefore, the EMT Initiative specialised modules must leverage the capacity of the AVoCH-SURGE core responders, who are provided with an additional and crucial operational arm. Additionally, the complementarity between Emergency Operations Centres (EOCs), Rapid Response Teams (RRTs), and EMTs in response to PHEs in the African context is key in enhancing response action; hence, it is a work in progress (43).

## 4 Lessons learnt

This section presents the lessons learnt from the journey of the EMT initiative in the WHO African Region. The results have been synthesised from document reviews and interviews and presented using components of a framework from Adini et al. (17).

### 4.1 Financing and operations supply and logistics (OSL)

*EMT concept was perceived as being finance, supplies, and logistics-intensive beyond the capacity of many countries.* During the initial workshops with the countries, when the concept was first presented, most countries highlighted their inability to meet the financial needs of attaining self-sufficiency with the idea, which included purchasing the requisite equipment and maintaining an able team of human resources. Many of the countries, at the onset, suggested that it would have been better to have national teams that were able to respond to an internal emergency for which the governments did not have to spend extra costs as they are already on the government payroll. Despite these challenges, the region’s focus has been on building national EMTs, though the need to attain self-sufficiency in technical and logistics capacity remains difficult.

the countries … suggested … to have national teams … for which the governments did not have to spend on their salaries because they would be considered part of the employee. The concept was supplies and equipment intensive, and many governments could not commit payments to achieve it…. The concept of the EMT is yet to be part of the continent's culture. (Respondent 04)

*Despite the perception of financing, the initial financial support (to the WHO AFRO for the concept) was a useful strategy because it provided the ability to work on the concepts, set up systems, and run programmes with the countries.* It brought confidence and autonomy in managing the EMT activities, such as the ability to hire personnel, e.g., a full-time consultant, whose full responsibility was implementing EMT work and ensuring continuity of the work.

### 4.2 Initiation and driving desire

*Before the onset of the workshops, initial global training on EMT injected some desire into the EMT concepts, and countries were eager to join and learn.* It brought an understanding of what EMTs are, which was quite useful. The initial concept was on the international classification of EMTs, which brought the idea of identifying two countries that wanted to join the international classification process.

However, when the concept was initially presented to the countries at its onset, it was perceived as something from the West (i.e., the global north to the global south) akin to colonial medicine. (Respondent 04)

The concept involved an international/external team coming to different African countries to support emergency response and work with the country’s counterparts. During the awareness workshops in the countries, the country teams often conflicted with many questions regarding whether they needed those teams to solve their problems.

*Countries that took up the concept in the initial awareness workshops were convinced of their usefulness because of their level of preparedness and the desire to be better, but also because of the workforce that was convinced it would be better based on their resources.* For instance, the Senegal Military was convinced that the concept would work because of their operational position and assets (such as aeroplanes and boats), making the work easier. Furthermore, at that time, someone was handling the PHEOC who was also quite positive and supported the creation of a good work dynamic. The Senegal army EMT, which was prepositioned at the commencement of the Initiative, picked it up well and has been working to respond to PHEs in the region. The army EMT concept has worked well because they are well equipped and have the government’s support.

In May 2022, Senegal’s military emergency medical team carried out a drill, with support from WHO, to bolster its capacity and obtain international certification for high standard of emergency response…. If Senegal is successful, it will become the first African country to obtain international classification. [WHO AFRO Article (46)]

### 4.3 Planning, regional, and global collaboration

*Conflict with other global and regional initiatives that were already or are trying to become well established.* In Africa, most of the PHE are linked to outbreaks. From the lessons on the response to Ebola, an idea to develop a kind of outbreak-oriented EMT was brought forth, as the initial concept of the EMT was mainly focussed on handling natural disasters. This suggestion, however, was not as well received.

As the EMT concept/Initiative was being implemented, there was a perceived conflict with the other WHO AFRO initiatives, such as the proposed AVoCH-SURGE project. However, it was proposed that the two needed to be linked as they were perceived to serve the same function but with a different governance.

*As the Initiative grows and there is a standardisation of activities, it would be imperative to bring in some of the diplomacy knowledge in the concept and include the support of the country’s Ministries of Foreign Affairs because they play a key role in permitting the arrival of I-EMTs.* The EMT concept, particularly in the region, is very geopolitical and requires a lot of tactical diplomacy, especially when there is the involvement of I-EMTs being deployed to the continent.

For instance, because of the language and cultural practices, it is plausible for a country like Mozambique to work well with Angola because of language and shared cultural aspects. However, the deployment experience has shown that this aspect of pairing teams, considering cultural knowledge, may have been missing. For example, as a coordinating person leading the Initiative, it would be easy to tell Angola that they're not EMTs and should not come to Mozambique. However, the governments guided by their shared sanitary diplomacy would enhance the deployment. (Respondent 03)

*The EMT values rely on standards and quality, which would be imperative to maintain; they bring uniformity about how responses are made, which is key.* Strategically, strong regional leadership and expanded multilateral partnerships (such as with African CDC and African heads of state) will enhance countries’ engagement and stronger implementation of the Initiative (47).

The EMT has good standards … they bring uniformity about how [emergency] response is made … positioning the future of the EMT work may require the team to consider working with the African CDC, which is more independent and closer to the African heads of state, to implement the Initiative. Given that the challenge of the EMT initiative is funding-driven, working with a body closer to the countries will work well to ensure acceptability and the quick implementation of the strategy. (Respondent 05)

### 4.4 Human resource challenges and opportunities

*There was difficulty changing the visions of the EMT to fit the context given the changes in the human resources.* There was rigidity that the initial EMT concept, whose description was about the subheading, needed to remain as it was, yet there were challenges.

For example, we wanted outbreak-oriented changes, but the new staff wanted to have type one, type two, and type three. (Respondent 01)

*Consistent leadership is imperative to enhance the EMTs’ work in the region.* With consistent messaging and the deployment of different steps, today, we have many countries interested in the concept and funding their teams for training and development. These newer country teams have been deployed regionally for further training in different countries and are even used to respond to local crises. For instance, Ethiopia has enhanced its national team with the government’s support, which has even seen it being deployed and partnered with other international groups through intergovernmental support. On the other hand, teams such as Namibia have been used to respond to cholera outbreaks in Malawi.

### 4.5 Training programmes and exercises and contextualising the concept

*Several outbreaks in the WHO African region bolstered the rollout of the EMTs.* For example, the measles outbreak, Ebola, explosion, COVID-19. The first deployment was a team from Senegal supported and capacitated under visionary leadership. The Senegal team took up the lessons from the awareness workshop and rolled with it quickly. They developed the SOPs within a very short period and started EMT work. After that, following an explosion in DRC, they were sent to respond and even came with equipment and materials. The Senegalese team provided support for 6–7 weeks and trained the locals on basic management. It was the initial lesson learned that this type of deployment was possible. They also supported the response in Mozambique, Sierra Leone, and the Gambia.

*The WHO Regional Office for Africa actively participated in global EMT activities to learn new ways of reshaping the EMT to fit the local context.* For instance, amidst the response work, the team contributed to developing work through the blue book and other similar documents.

*COVID-19 brought a new way of thinking in the EMT deployments and changed the concept to fit the thinking.* With COVID-19 came more money and leadership keen to enhance the focus of EMT work. The outbreak also provided an opportunity to improve the deployments for teams in different countries. It further brought rise to the concept of developing a training centre, helped to advance the 10 steps of creating an EMT, and even supported the clinical and organisational management of the EMTs.

*Timeliness is needed over perfection in the EMT work, particularly in the African Continent.* Lessons from using the EMTs in the Ebola crisis showed that the I-EMTs would take too long to set up ideal triage settings meeting the standards set (for instance, they would do a working triage process and flow that would take 1 month to build). On the contrary, when the team hired local Africans to make a triage setup, it was set up in half a day using locally available materials, giving them more time to focus on working with the patients. There is a need for flexibility in how things are done.

It will be imperative to contextualise the EMT concept to consider our unique characteristics (language and culture) moving forward. The continent has a lot of capacity for science and medical expertise that can be leveraged to build stronger EMTs.

## 5 Discussion

This article describes the progress of the WHO African Region in implementing the EMT Initiative from 2015 to December 2023. It discusses significant milestones and challenges, offering valuable insights into the implementation process within the African context. The journey, guided by stages and actions based on the SIC framework, emphasises the importance of considering the specific context and global emergency response lessons.

Our results showed the origin of the global EMT concept and how the continent’s challenges shaped the introduction of EMT in the WHO African Region. Furthermore, the results have shown the initiation of regional sensitisation workshops and country awareness campaigns on the concept and how the countries bought in the idea and agenda. There was a need to institute the EMT initiative in the region to enhance the timeliness of response and quality of service provided during emergencies whilst enhancing the national/country capacities to respond. Being a region experiencing more than 100 emergencies at any given time (48), with a lack of classified teams to timely respond to the different, in some cases context-specific challenges of the African countries, the WHO prioritised the African Continent as a prime for the EMT concept. In addition, the continent faces health emergencies yearly emanating from climate-related events (such as protracted droughts, calamitous floods, and cyclones), human–animal–environmental interface, and prolonged humanitarian crises (such as cross-border movements, internal population displacements, and mass refugee migration) (48–52) which the EMT concept was envisioned to help to respond to.

Furthermore, the study has shown that the initial phase (2015–2017) focussed on engagement, feasibility testing, and readiness planning, which highlighted the unique challenges faced by the African region and required a tailored approach to the EMT concept. The initial global EMT principles, which focussed primarily on trauma and surgical care for sudden-onset disasters (SOD), needed to be broadened, and the establishment of the WHO Health Emergencies (WHE) Programme provided a structured framework for the Initiative.

The EMT initiative faced several challenges during its implementation in the African region. The region’s unique health system vulnerabilities, resource limitations, and the complexity of coordinating multiple stakeholders posed significant hurdles. In addition, the pandemic exacerbated the existing challenges to the fragile health system, coupled with multiple emergencies happening in most countries, and hampered the continuity of health services in the region (2, 53). However, these challenges also provided valuable lessons for future emergency response planning. The key themes identified from the implementation process include the importance of local context adaptation, capacity building, stakeholder engagement, and sustainable funding mechanisms.

The EMT initiative benefited from several outbreaks, such as Ebola and COVID-19, underscoring the importance of timely and flexible responses. The Initiative has demonstrated that local adaptations, such as using local materials for triage setups, can significantly enhance efficiency. This flexibility and local adaptation are also emphasised in EMT deployments in the Pacific region, where local practices and materials are often utilised to great effect (54). Adapting the EMT concept to fit local contexts whilst maintaining consistent leadership and messaging has been critical (55). Countries like Ethiopia and Namibia have successfully developed national teams with government support and have participated in regional responses. This highlights the importance of context-specific adaptation and consistent leadership, similar to the experiences in South Asia, where consistent leadership and local adaptation have been key to successful EMT deployment (56).

The global training on EMTs initially injected enthusiasm into the concept, but some perceived it as an external imposition reminiscent of colonial medicine. Nonetheless, countries like Senegal demonstrated the concept’s viability, leveraging their operational assets and government support to develop effective EMTs. This mirrors experiences where strong national support and pre-existing military structures facilitated EMT implementation (38, 57–60), and training programmes enhanced it (61). As such, the WHO African Region fortified the elements of the training by developing a training centre that was positioned to enhance and fortify the technical skills of regional EMT members, including the N-EMTs, provide specialised training and clinical care management (such as in managing severely sick and critical patients), initiate healthcare workers to enhance operational hands-on skills and act as an EMT innovation centre in the WHO African Region. At the onset, the initial training curriculum was envisioned to be developed with the support of the other WHO EMT networks, other WHO technical units, associated WHO collaborating centres, and members of academia.

Following the progress of the concept in the region, it has highlighted how the countries use the skills acquired to conduct regional and local deployments, which they use to enhance the development of skills and knowledge transfer. Furthermore, it has highlighted the development of the WHO AFRO strategy and course of action for the future, including establishing and prioritising the development of the N-EMTs, development of the roadmap of implementation, the proposed coordination, and the regional training centre as crucial elements of the EMT strategy in the region. These align with the aims and objectives of the global EMT 2030 strategy, which envisions a globe where every country can respond effectively and timely to national PHEs, leveraging on the regional and sub-regional capacities to help vulnerable communities and others in need but also strengthening information systems, evidence and research (62). The lessons learnt show a need to work on additional components that can improve the work. For instance, there is a need to establish a framework for collaboration with I-EMTs which is context-specific and diplomatically sound for the countries. Such a partnership would enhance the deployment and procurement modalities. In addition, working on the complementarity between other regional initiatives would be imperative.

From the lessons learnt, the EMT concept was perceived as financially and logistically intensive, beyond the capacity of many countries in the region. Initial workshops highlighted countries’ inability to meet the financial needs for self-sufficiency, including purchasing equipment and maintaining human resources. Despite this, the initial financial support to WHO AFRO was crucial in setting up systems and running programmes, thereby building confidence and autonomy in managing EMT activities. Similar challenges have been observed in other regions where financial constraints hinder EMTs’ sustainability (63, 64).

The EMT initiative faced conflicts with other regional initiatives, such as the AVoCH-SURGE project. Effective integration and collaboration with these initiatives and with bodies like the African CDC are essential for harmonised and efficient emergency response. This is comparable to the European experience, where integrating EMTs with existing emergency frameworks has been crucial for success (65).

## 6 Conclusion

The EMT initiative in the African region from 2015 to 2023 has been a flexible and adaptive process shaped by global insights and regional obstacles. The engagement, feasibility testing, and readiness planning phases have highlighted the significance of tailored approaches and collaborative efforts to strengthen emergency preparedness and response capabilities. The journey of the Initiative has provided valuable lessons in financing, initiation, collaboration, human resources, and training. Each area has emphasised the importance of context-specific adjustments, robust regional and national support, and flexible, timely responses. Compared with experiences from other regions, these lessons demonstrate common challenges and strategies, highlighting the global relevance of these insights. As the Initiative moves forward, continuous learning, adaptation, and investment in local capacities will be crucial for establishing resilient health systems capable of effectively addressing future emergencies. Furthermore, the continuous refinement of the EMT initiative is crucial. Emphasis should be placed on strengthening local health systems, enhancing training and capacity-building programmes, and fostering regional and international collaborations. Additionally, sustainable funding and resource allocation are essential to ensure the resilience of EMTs in the African region and their long-term success.

## Data availability statement

The original contributions presented in the study are included in the article/supplementary materials, further inquiries can be directed to BO oyugib@who.int and the corresponding author.

## Ethics statement

Ethical approval was not required for the study involving humans in accordance with the local legislation and institutional requirements. The studies were conducted in accordance with the local legislation and institutional requirements. Written informed consent to participate in this study was not required from the participants in accordance with the national legislation and the institutional requirements. The participants provided their verbal informed consent to participate in this study.

## Author contributions

TB: Funding acquisition, Resources, Supervision, Writing – original draft, Writing – review & editing. BO: Conceptualization, Data curation, Formal analysis, Investigation, Methodology, Writing – original draft, Writing – review & editing. J-JM: Data curation, Formal analysis, Writing – original draft, Writing – review & editing. RK: Data curation, Formal analysis, Writing – original draft, Writing – review & editing. LM-M: Data curation, Formal analysis, Writing – original draft, Writing – review & editing. PR: Conceptualization, Data curation, Formal analysis, Methodology, Writing – original draft, Writing – review & editing. CL: Conceptualization, Data curation, Formal analysis, Investigation, Methodology, Writing – original draft, Writing – review & editing. DB: Data curation, Formal analysis, Investigation, Methodology, Writing – original draft, Writing – review & editing. AF: Funding acquisition, Resources, Supervision, Writing – original draft, Writing – review & editing. JO: Resources, Supervision, Writing – original draft, Writing – review & editing. FS: Conceptualization, Formal analysis, Methodology, Software, Writing – original draft, Writing – review & editing. FB: Supervision, Writing – original draft, Writing – review & editing. DC: Funding acquisition, Supervision, Writing – original draft, Writing – review & editing. AG: Funding acquisition, Resources, Supervision, Writing – original draft, Writing – review & editing. N'DY: Funding acquisition, Resources, Supervision, Writing – original draft, Writing – review & editing. IF: Funding acquisition, Resources, Supervision, Writing – original draft, Writing – review & editing.
